# An Epigenetic Locus Associated with Loss of Smell in COVID-19

**DOI:** 10.3390/diagnostics14242823

**Published:** 2024-12-15

**Authors:** Elif Sibel Aslan, Kenneth White, Gulsen Meral, Zeyneb Nur Akcay, Aytug Altundag, Savas Gur, Mehmet Dokur, Mehmet Akif Baktir, Lutfiye Karcioglu Batur

**Affiliations:** 1 Department of Molecular Biology and Genetics, Biruni University, Merkezefendi, 75 Sk No:1-13 M.G., 34015 Istanbul, Turkey; 2 Biruni University Research Center (B@MER), Biruni University, 34015 Istanbul, Turkey; 3 School of Human Sciences, London Metropolitan University, 166-220 Holloway Road, London N7 8DB, UK; 4 President’s Office, Department of Pediatrics, Nutrigenetics and Epigenetics Association, 34000 Istanbul, Turkey; gulsenmeral@drgulsenmeral.com; 5 Epigenetics Coaching, 262a Fulham Road, Suite 1, First Floor, Chelsea, London SW10 9EL, UK; 6 Department of Otorhinolaryngology, Biruni University, Merkezefendi, 75 Sk No:1-13 M.G., 34015 Istanbul, Turkey; aaltundag@biruni.edu.tr; 7 Independent Researcher, 17000 Çanakkale, Turkey; drsavasgur@gmail.com; 8 Department of Emergency Medicine, Faculty of Medicine, Bilecik Seyh Edebali University, 11230 Bilecik, Turkey; drdokur@gmail.com; 9 Department of Physiology, School of Medicine, Erciyes University, 38030 Kayseri, Turkey

**Keywords:** SARS-CoV-2, UGT1A1, olfaction, anosmia, epigenomics, DNA methylation

## Abstract

Background/Aim: Loss of smell, also known as anosmia, is a prevalent and often prolonged symptom following infection with SARS-CoV-2. While many patients regain olfactory function within weeks, a significant portion experience persistent anosmia lasting over a year post-infection. The underlying mechanisms responsible for this sensory deficit remain largely uncharacterized. Previous studies, including genome-wide association studies (GWAS), have identified genetic variants near the UGT2A1 and UGT2A2 genes that are linked to anosmia in COVID-19 patients. However, the role of epigenetic changes in the development and persistence of smell loss has not been well explored. In this study, we aimed to investigate epigenetic alterations in the form of DNA methylation in the UGT1A1 gene, which is a locus associated with olfactory dysfunction in COVID-19 patients. Methods: We analysed DNA methylation patterns in blood samples from two carefully matched cohorts of 20 COVID-19 patients each, which are differentiated by their olfactory function—those with normal smell (normosmia) and those suffering from smell loss (anosmia). The cohorts were matched for age and sex to minimize potential confounding factors. Results: Using quantitative analysis, we found significantly lower levels of DNA methylation in the UGT1A1 locus in the anosmia group compared to the normosmia group, with a 14% decrease in median methylation values in patients with smell loss (*p* < 0.0001). These findings highlight potential epigenomic alterations in the UGT1A1 gene that may contribute to the pathogenesis of anosmia following COVID-19 infection. Our results suggest that the methylation status at this locus could serve as a biomarker for olfactory dysfunction in affected individuals. Conclusion: This study is among the first to describe epigenetic changes associated with smell loss in COVID-19, providing a foundation for future research into targeted interventions and potential therapeutic strategies aimed at reversing persistent anosmia. Further investigations involving larger cohorts and additional loci may help elucidate the complex interaction between genetic, epigenetic, and environmental factors influencing long-term sensory impairment post-COVID-19.

## 1. Introduction

Loss of smell, a common symptom experienced by many individuals infected with COVID-19, has garnered significant attention due to its high prevalence and prolonged persistence in some patients [1,2,3]. This olfactory dysfunction can manifest as a complete loss of smell (anosmia) or partial reduction in olfactory sensitivity (hyposmia), which is in contrast to normal olfactory function (normosmia). Self-reported studies estimate that 35–50% of COVID-19 patients experience some degree of smell loss [3]. While the exact cause of olfactory dysfunction following SARS-CoV-2 infection remains unclear, current evidence suggests that it primarily involves damage to the olfactory epithelium, which expresses high levels of viral receptors such as ACE2 and TMPRSS2. In contrast, olfactory sensory neurons (OSNs), responsible for transmitting smell signals to the brain, do not appear to be directly infected by the virus [1,3,4,5,6]. This epithelial damage likely leads to inflammation and disruption of the local microenvironment, which in turn affects olfactory function.

Olfactory dysfunction following viral infections is not unique to COVID-19. Persistent anosmia can also result from upper respiratory tract infections, head trauma, and nasal or sinus disorders such as chronic rhinosinusitis. Less common causes include environmental exposure to harmful chemicals and medical procedures like surgery, radiation, or chemotherapy, which can have long-term effects on olfactory pathways [7]. Though loss of smell may seem like a minor inconvenience, it has profound implications for an individual’s quality of life. The ability to smell is intricately linked with enjoying food, detecting personal hygiene issues, and maintaining overall well-being. Studies have shown a connection between olfactory dysfunction and increased rates of depression, anxiety, and social withdrawal due to the inability to appreciate scents and flavours, leading to emotional distress and isolation [8]. Additionally, olfaction plays a critical role in safety, allowing individuals to detect hazards such as smoke, toxic chemicals, or spoiled food. Impaired olfaction could, therefore, indirectly lead to life-threatening situations, making the issue more significant than initially perceived [9].

Interestingly, there is also evidence to suggest a link between olfactory health and overall mortality rates. Individuals with a strong sense of smell have been observed to have lower mortality rates, likely due to their heightened awareness of their environment and potential dangers, though the mechanisms underlying this association are not fully understood [8]. This further underscores the importance of olfaction beyond its role in sensory perception. Given the extended persistence of olfactory dysfunction in some COVID-19 patients, with cases lasting for over a year after initial infection, it becomes imperative to thoroughly investigate the underlying causes and mechanisms of smell loss [10]. Understanding how SARS-CoV-2 leads to this dysfunction at both the cellular and molecular levels may help identify therapeutic targets and develop treatments to restore olfactory function. Research into the long-term effects of COVID-19 on sensory systems, particularly the olfactory system, is crucial as healthcare providers and scientists continue to grapple with the wide-ranging and sometimes lingering impacts of this novel virus. Furthermore, exploring potential epigenetic, genetic, or immunological factors that may predispose certain individuals to persistent anosmia could provide critical insights into both the prevention and treatment of COVID-19-related olfactory dysfunction.

While environmental differences could account for the variation in olfactory dysfunction observed across COVID-19 patients, genetic background could also be an important factor and was investigated in a genome-wide association study (GWAS) involving 70,000 COVID-19 patients [11]. A locus including the UGT2A1/UGT2A2 gene was strongly associated with loss of smell across all ancestries sampled, including European, African, South Asian, and East Asian [11]. UGT2A1 and UGT2A2 are part of a family of uridine diphosphate glycosyltransferases, which includes UGT1A1, that conjugate lipophilic substrates with glucuronic acid to enhance their excretion. Studies in rats show that these enzymes, which are expressed in the olfactory epithelium, are involved in the elimination of the odorants that enter the nasal cavity and bind to olfactory receptors [12]. A crucial question is how these genes can affect olfaction following infection with SARS-CoV-2. The index mutation of the locus identified in UGT2A1/UGT2A2 is speculated to enhance olfactory sensitivity such that people carrying the mutation will be more likely to experience a loss of smell after infection [11]. The mechanism by which gene function can be altered after viral infection is not known. One possibility is an epigenetic change. There are no studies we are aware of that examine methylation changes in olfactory genes following infection by SARS-CoV-2. In this report we describe the first identification of epigenetic differences associated with SARS-CoV-2 infection linked to anosmia. We selected UGT1A1 as a target because no SNP variants associated with anosmia have been identified in UGT1A1 and it is a homolog of UGT2A1 implicated in olfactory function [13].

## 2. Materials and Methods

### 2.1. Patients

Patients were invited to participate and informed consent was obtained. The clinical data of patients with a laboratory-confirmed COVID-19 infection were obtained from the University of Biruni Hospital. The following inclusion criteria were used for this study: patients with mild to moderate COVID-19 who did not require intensive care, participants aged 17 years or older, and patients that had a laboratory-confirmed COVID-19 infection as determined by RT-PCR testing, were native speakers, and were independently able to complete the study questionnaire and olfactory testing. Specific symptoms or clinical presentation of the disease were not taken into account as inclusion criteria. Exclusion criteria included a history of olfactory or gustatory dysfunction prior to the outbreak, a lack of a laboratory-confirmed COVID-19 infection, and being in the intensive care unit at the time of the study. All patients who met the inclusion criteria were initially qualified for analysis. If any patients were excluded, it was due to factors such as incomplete data, an inability to perform the required olfactory tests, or failure to meet other inclusion criteria. No specific patient characteristics were presented, ensuring the focus remained on the methodology and results. Patients were recruited from the early 2020 period of the pandemic, when the original Wuhan strain and its early mutations were dominant. Patients initially self-reported olfactory sensitivity, which was then assessed within two weeks of diagnosis of a SARS-CoV-2 infection using odorants presented in commercially available felt-tip pens (“Sniffin’ Sticks” Burghart GmbH, Wedel, Germany) [14]. Olfactory testing used three tests: tests for odour threshold T (testing by means of a single staircase procedure), door discrimination D (3-alternative forced choice), and odour identification I (4-alternative forced choice) [14]. Scores from the three tests were combined into a TDI score. Patients selected were either normosmic (TDI > 30) or anosmic (TDI < 16) [15,16]. 

### 2.2. DNA Extraction

Following diagnosis of SARS-CoV-2 and inclusion into the study, blood samples were collected in EDTA tubes and DNA extracted from whole blood samples using the Zymo Research Quick-DNA Miniprep Plus Kit (D4068, Irvine, CA, USA), following the manufacturer’s protocol specific to biological fluids. After completing the isolation process, the DNA was eluted with 50 µL of DNase/RNase-free water. Concentration and purity (A260/A280 ratio) were measured using a Nanodrop spectrophotometer (Thermo Fisher Scientific, Waltham, MA, USA). To avoid bias in measurements due to differences in DNA concentration, the concentration of DNA from each sample was normalized to 4 ng/µL by dilution. This step is critical for ensuring consistency in downstream analyses, such as methylation studies, where precise DNA concentrations are required to achieve reliable and reproducible results. The use of this high-quality DNA extraction method ensures that the samples are suitable for subsequent molecular analyses, such as epigenetic profiling or gene expression studies.

### 2.3. Methylation Analysis

Methylation at the target locus was assessed by qPCR using a Zymo Research OneStep qMethyl-PCR Kit (D5310). A total of 5 µL (20 ng) of the isolated DNA samples were added to 10 µL of Test or Reference Mix containing methylation sensitive restriction endonucleases (MSREs), 2 µL of Primer Mix, and 3 µL of DNAse/RNAse Free Water, according to the protocol of the kit. The samples were analysed using a BMS Mic qPCR apparatus together with methylated and unmethylated DNA standards present in the Reference Mix. For each sample a reference qPCR analysis was compared with a test analysis in which DNA was pre-digested with MSREs. The percent methylation across the target region was calculated according to the equation:

% Methylation = 100 × 2^−ΔCt^

where ΔCt = Average Ct of test reaction—Average Ct of reference reaction.

Methylation maps of the UGT1A1 locus were obtained by downloading an epigenomics metadata file from NCBI (https://www.ncbi.nlm.nih.gov/epigenomics accessed on 7 January 2023). Data for samples of white blood cells were visualised using the Roadmap Epigenomics Program data at the gene expression omnibus (GEO) (https://www.ncbi.nlm.nih.gov/geo/roadmap/epigenomics accessed on 2 January 2023); GEO files of DNA methylation for peripheral blood monocytes GSM613911 and GSM669606 were used.

### 2.4. UGT1A1 Locus

The locus used is located towards the 3′ end of the intron in the gene UGT1A1. An amplicon of 231 bp, chr2:233766213-233766443, was generated from the forward primer AAG-GGG-ATG-GAA-TGG-GAA and reverse primer AGA-CAC-ACA-GGT-AGC-TGG-AC. The sequence of the locus, methylation sites, and SNP loci were obtained from the WashU Epigenome Browser (https://epigenomegatewya.wustl.edu/browser accessed on 6 January 2023). SNP allele frequency was obtained from dbSNP (https://www.ncbi.nlm.nih.gov/snp accessed on 1 February 2023).

**Figure 1:**
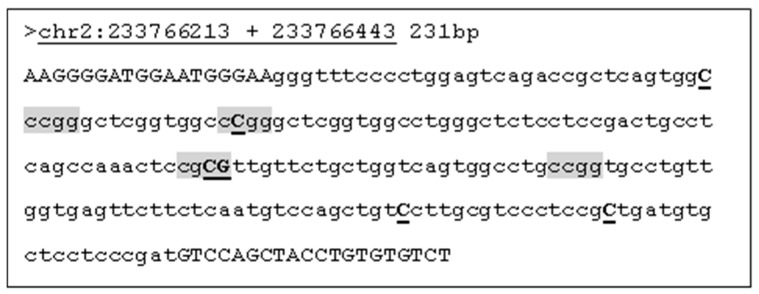
Locus of UGT1A1 analysed. Primers are written in upper case. Methylation sites are shaded in grey. Positions of SNPs are shown in upper case, bold, and underlined. The SNP rs4663334 is located in the second methylation site shown.

The amplicon and methylation sites analysed in this study are shown in Figure 1, together with positions of the SNPs found in the locus. Three SNPs coincide with methylation sites, one of which, rs4663334, has a global allele frequency of 0.137 (Table 1) and could potentially affect levels of DNA methylation within the locus analysed. The other two SNPS have global allele frequencies of <0.001 and can be ignored. A list of the SNPs and mean allele frequencies are given in Table 1.

**Figure 2:**
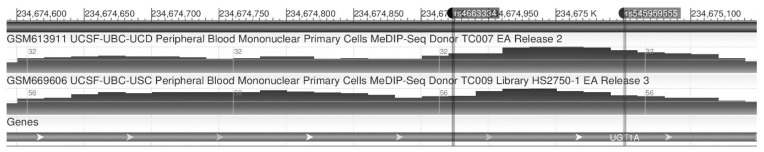
Methylation profiles of mononuclear blood cells in the UGT1A1 locus. The top scale shows the position of the locus in chromosome 2 and the positions of SNPs rs4663334 and rs545959455. The methylation landscape for two analyses of blood mononuclear cells across the locus of UGT1A1 are shown in the second and third scales.

Since the methylation analysis was to be carried out on DNA extracted from white blood cells, we checked the NCBI GEO Epigenomics database for methylation profiles of representative white blood cell populations to confirm that methylation had been documented in the UGT1A1 locus selected for this study. We could find methylation profiles for mononuclear cells (Figure 2), which were obtained using MeDIP-Seq methodology [17] and indicated about 50% methylation at the chosen locus. This suggested that the locus was suitable for assaying measurable changes in methylation.

### 2.5. Statistical Analysis

Data were analysed using GraphPad Prism 9.5.1. Normality of the data was assessed using Shapiro–Wilk and Kolmogorov–Smirnov tests. Non-parametric tests were used when normality tests were not passed. The threshold for significance was *p* < 0.05. Individual tests are described for each data set.

## 3. Results

### UGT1A1 Locus Methylation in COVID-19 Patients

In this study DNA from 20 patients with COVID-19 who had not lost their sense of smell was analysed in comparison with DNA from 20 patients with COVID-19 who had lost their sense of smell for at least one year.

There is no significant difference between the two groups (Table 2) in age (*p* = 0.379; unpaired t test with Welch’s correction) or sex (*p* = 0.428; Fisher’s exact test). There was no difference in the distribution of ages across the two groups, as indicated by a comparison of fits of the curves for normosmic vs. anosmic by least squares regression (*p* = 0.095) between the data sets.

**Figure 3:**
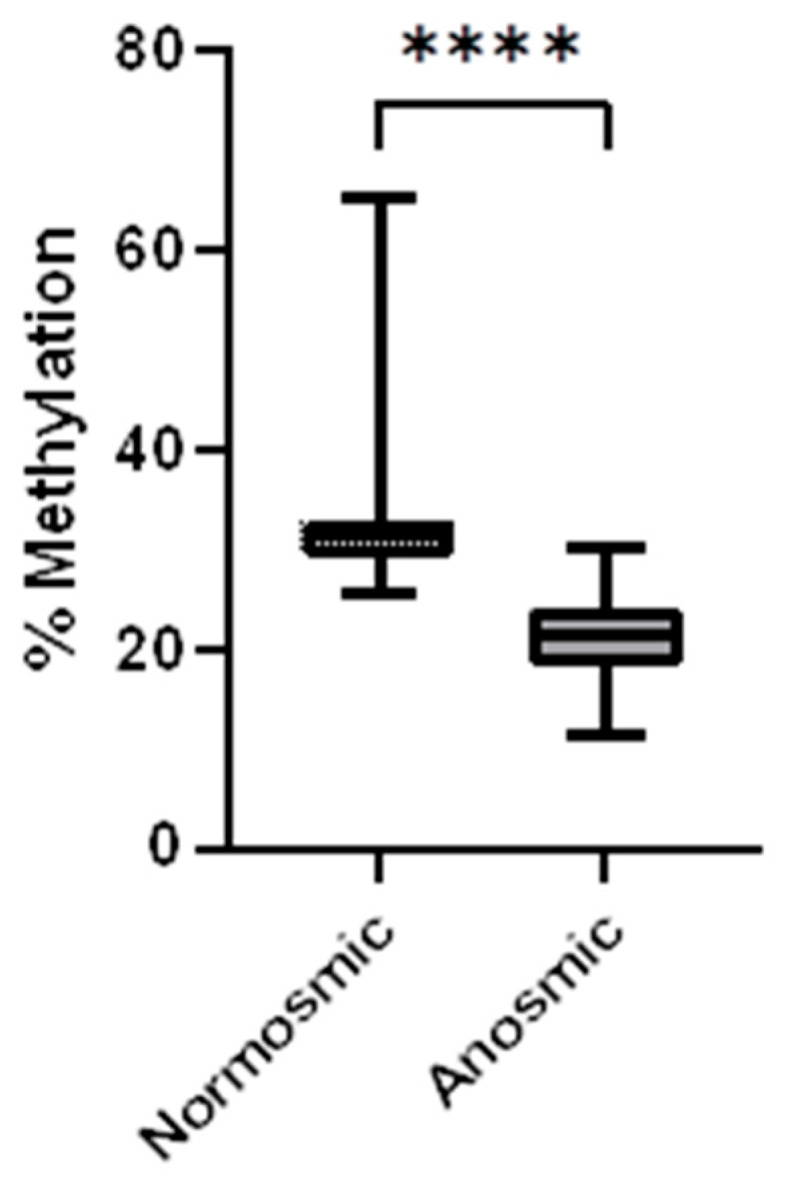
Comparison of methylation in the UGT1A1 locus. Median, quartiles, and ranges are shown. The mean methylation was 35.3% and 21.0% for the normosmic and anosmic groups, respectively (**** *p* < 0.0001).

Methylation in the UGT1A1 locus for each patient is shown in Table 3 and differences between the two groups are shown in Figure 3. The median % methylation in normosmic COVID-19 patients, 31.3%, is significantly higher than in anosmic COVID-19 patients with loss of smell, 21.4% (Mann–Whitney test *p* < 0.0001).

**Figure 4:**
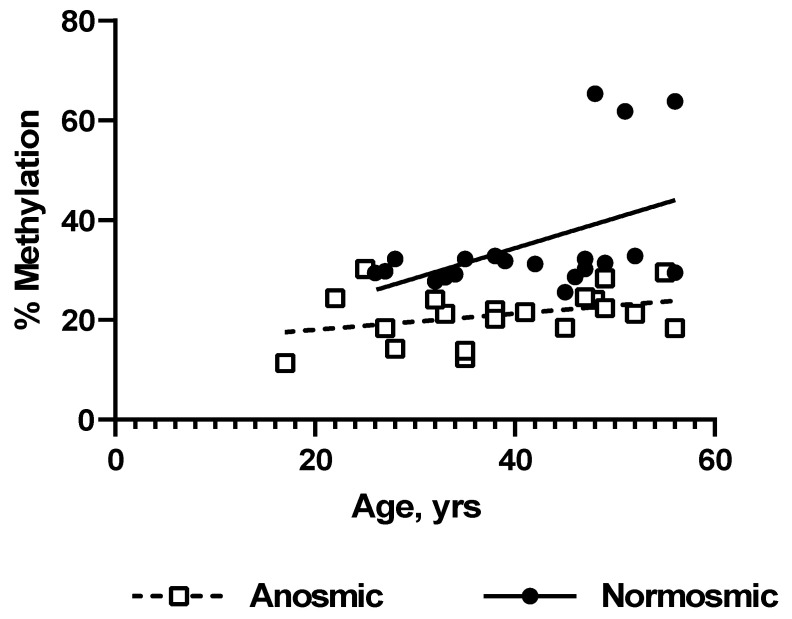
Correlation analysis between methylation in the UGT1A1 locus and age.

We analysed the correlation between smell loss and age by simple linear regression, as shown in Figure 4. In the anosmic group there was no correlation (R^2^ = 0.118, *p* = 0.139), but in the normosmic group there was a slight correlation (R^2^ = 0.214, *p* = 0.040), which was lost when the three outliers were excluded (R^2^ = 0.009, *p* = 0.717). Larger sample sizes are needed to confirm the lack of correlation between methylation and age suggested by the data.

## 4. Discussion

In this study we present data suggesting the possibility that DNA methylation in blood cells could be a marker for loss of smell. A significant decrease in methylation of the locus tested, towards the end of intron 1 in the gene UGT1A1, was found in COVID-19 patients who had lost their sense of smell (Figure 3). The two cohorts of COVID-19 patients tested were small but had similar age distributions (Table 2 and Table 3, Figure 3), and the differences between the groups are not attributable to differences in age or sex (Table 2). Despite the small size of the cohorts, the difference in methylation levels was robust (Figure 3, *p* < 0.0001) and is readily discernible by visual inspection of the individual data points in Figure 4. The difference in mean methylation levels between the cohorts is 14% (anosmic mean ± SD 21.1 ± 5.3%, normosmic 35.3 ± 12.4%)

The locus was selected to have measurable changes in methylation that could be monitored. By choosing a locus with estimated mid-range levels of methylation, both decreases and increases could potentially be identified. One of the methylation sites contains the SNP rs4663334, in which C is mutated to T, with a global mean allele frequency of about 14% and a range of 11.6–23% across all populations. It is possible that some of the difference observed between groups could be due to the presence of the SNP, which would reduce methylation. A more thorough analysis should include genotyping each sample to assess the potential impact of genetic background which could either directly affect methylation through mutation of the methylation site or have an indirect effect as methylation quantitative trait loci [17]. This could be achieved over a longer locus or many loci to improve sensitivity, using nanopore single strand DNA sequencing technology that can simultaneously detect methylated bases [18]. In cases of post-COVID anosmia, the observation that the UGT1A1 locus remains hypomethylated, which could suggest a potential role of epigenetic mechanisms. This finding led us to consider a secondary question: as we recognize the significance of SAM as a one-carbon donor in DNA methylation, could the variation in individuals’ SNPs be responsible for differences in the enzymes involved in the methionine cycle. An investigation of genetic background could include loci relevant to this pathway.

The molecular link between viral infection and changes in DNA methylation remains elusive. Several studies, summarized by Ozturkler and Kalkan, 2021 and Krause et al., 2024, describe changes in DNA methylation in the ACE2, the cellular receptor for SARS-CoV-2 [19,20], and other key genes such as TMPRSS2, IFN-related genes, and FURIN. All are important determinants of SARS-CoV-2 cell entry into the host and of severity of the disease. The ability of the SARS-CoV-2 to modulate gene expression epigenetically may arise from the several proteins in the viral genome involved in RNA expression and modulation, especially the non-structural proteins (NSPs) [21]. Expression of viral miRNAs and modification of RNA are possible mechanisms, but it is not clear how these could link to modifications of DNA.

The functional interpretation of the findings is challenging. There has been a report linking methylation changes in intron 1 of genes to gene expression [22]. (The protein expressed by UGT1A1 belongs to the UDP-glucosyltransferase family, which includes UGT2A1/2A2. It is expressed in nasal epithelium, but not as much as UGT2A1/2A2, and both genes are expressed in blood (UniProt Consortium 2023 [23]; accession numbers P22308 (UGT1A1), P0DTE4 (UGT2A1)), which is the sample type analysed in this study. Epigenome wide association studies look for an association between tissue methylation patterns of DNA and disease states and are mostly based on analysis of whole blood [24]. For example, blood-based EWAS have revealed loci as candidate markers for the risk of developing type 2 diabetes. However, methylation patterns in blood may not reflect patterns in distal tissue [24]. Whatever the link between smell and changes in methylation of genes in blood cells, the question arises by what mechanism could this occur. Is it a result of infection of blood cells by SARS-CoV-2 or a downstream manifestation of infection, for example, caused by changes in cytokine profiles during infection.

The results of this study indicate that epigenomic changes, which can be readily detected in blood samples, may hold significant diagnostic potential for monitoring loss of smell in COVID-19 patients. This finding opens up intriguing possibilities for using these epigenetic markers, not only for tracking smell loss in the context of SARS-CoV-2 infection, but also as a general diagnostic tool for olfactory dysfunction across various causes. However, to determine whether these changes are specifically associated with COVID-19 or represent a broader marker for smell loss, further investigation is required. In particular, examining epigenomic changes in olfactory tissue would be essential to establish a direct link between these modifications and the mechanisms of olfactory impairment. Additionally, conducting longitudinal studies could shed light on the persistence and stability of these epigenetic alterations over time and their correlation with the degree of smell dysfunction, potentially providing valuable insights into both the prognosis and potential reversibility of smell loss. Such research could pave the way for developing targeted interventions and therapeutic strategies aimed at restoring olfactory function in patients affected by both COVID-19 and other conditions that impair the sense of smell.

## 5. Conclusions

This study demonstrates a significant association between reduced DNA methylation at the UGT1A1 locus and persistent anosmia in COVID-19 patients. Using a well-defined cohort of mild to moderate COVID-19 cases from Biruni Hospital, we found a 14% decrease in methylation levels in anosmic patients compared to normosmic patients, with statistical significance (*p* < 0.0001). These findings suggest that hypomethylation at this locus may serve as a potential biomarker for olfactory dysfunction in the context of SARS-CoV-2 infection.

While this research provides novel insights into the epigenetic mechanisms underlying anosmia, it is limited by the small cohort size and the lack of information on SARS-CoV-2 variants. Further studies are needed to validate these findings across larger, more diverse populations and to explore whether these epigenetic changes are specific to SARS-CoV-2 or extend to other upper respiratory tract infections. Additionally, the functional consequences of hypomethylation in the UGT1A1 locus should be investigated to better understand its role in olfactory dysfunction.

This work establishes a foundation for future research into the epigenetic regulation of anosmia and highlights the potential of DNA methylation as a non-invasive biomarker for tracking and diagnosing olfactory dysfunction in COVID-19 patients.

## Figures and Tables

**Table 1:** SNPs and Global Mean Allele Frequencies (MAFs) in the *UGT1A1* locus analysed.

SNP	Variant	Position	Global MAF
rs34353734	C/T	2:233766262	0.002
rs4663334	C/T	2:233766278	0.137
rs192391946	C/A/T	2:233766326	<0.001
rs373926461	G/A/T	2:233766327	<0.001
rs11679312	C/T	2:233766390	0.007
rs545959555	C/T	2:233766405	<0.001

**Table 2:** Gender and age of patients studied.

	Gender M/F	Age, Yrs	
		Range	Mean ± SD
**Normosmic**	6/14	17–56	38.6 ± 11.4
**Anosmic**	4/16	26–26	41.6 ± 9.5

**Table 3:** % Methylation for each patient.

Normosmic			Anosmic		
Age	Sex	% Methyln	Age	Sex	% Methyln
42	Female	33.8	35	Female	28.7
48	Female	30.6	49	Male	20.3
34	Female	34.5	48	Female	21.7
32	Female	38.9	17	Male	14.3
35	Male	30.1	41	Female	18.7
26	Male	30.1	38	Female	22.0
47	Male	39.2	33	Female	15.6
49	Female	35.1	28	Female	23.3
28	Female	29.0	45	Male	18.4
52	Female	34.0	47	Male	19.9
56	Female	29.0	49	Female	18.4
51	Female	29.0	52	Female	30.7
45	Female	32.5	56	Female	21.3
33	Female	21.7	38	Male	16.1
38	Female	22.7	35	Female	25.5
27	Female	28.5	32	Male	15.3
56	Female	26.2	22	Female	22.4
46	Male	32.9	27	Female	11.6
39	Female	26.8	25	Female	14.8
47	Female	24.2	55	Female	22.4

## Data Availability

Enquiries for access to data should be addressed to Elif S Aslan, easlan@biruni.edu.tr.
